# Effects of Low-Load Blood Flow Restriction Training on Muscle Anabolism Biomarkers and Thrombotic Biomarkers Compared with Traditional Training in Healthy Adults Older Than 60 Years: Systematic Review and Meta-Analysis

**DOI:** 10.3390/life14030411

**Published:** 2024-03-20

**Authors:** Raúl Fabero-Garrido, Miguel Gragera-Vela, Tamara del Corral, Marta Hernández-Martín, Gustavo Plaza-Manzano, Ibai López-de-Uralde-Villanueva

**Affiliations:** 1 Department of Radiology, Rehabilitation and Physiotherapy, Faculty of Nursing, Physiotherapy and Podiatry, Complutense University of Madrid, 28040 Madrid, Spain; rfabero@ucm.es (R.F.-G.); mgrage01@ucm.es (M.G.-V.); gusplaza@ucm.es (G.P.-M.); ibailope@ucm.es (I.L.-d.-U.-V.); 2 Instituto de Investigación Sanitaria del Hospital Clínico San Carlos (IdISSC), 28040 Madrid, Spain; 3 Department of Nursing, Faculty of Nursing, Physiotherapy and Podiatry, Complutense University of Madrid, 28040 Madrid, Spain; martamah@ucm.es

**Keywords:** older adults, blood flow restriction therapy, muscle proteins, biomarkers, resistance training, review

## Abstract

The aim of this meta-analysis was to determine the effects of low-load blood flow restriction training (LL-BFRT) on muscle anabolism and thrombotic biomarkers compared with the effects of traditional LL training and to analyse the changes in these biomarkers in the short and medium term (acute/immediate and after at least 4 weeks of the training programme, respectively). A search was conducted in the following electronic databases from inception to 1 March 2024: MEDLINE, CENTRAL, Web of Science, PEDro, Science Direct, CINHAL, and Scopus. A total of 13 randomized controlled trials were included, with a total of 256 healthy older adults (mean (min–max) age 68 (62–71) years, 44.53% female). The outcome measures were muscle anabolism biomarkers and thrombosis biomarkers. The standardized mean difference (SMD) was calculated to compare the outcomes reported by the studies. The overall meta-analysis showed that LL-BFRT produces a large increase in muscle anabolism biomarkers compared with traditional LL training (eight studies; SMD = 0.88 [0.39; 1.37]) and compared with a passive control (four studies; SMD = 0.91 [0.54; 1.29]). LL-BFRT does not produce an increase in thrombotic biomarkers compared with traditional LL training (four studies; SMD = −0.02 [−0.41; 0.36]) or compared with a passive control (two studies; SMD = 0.20 [−0.41; 0.80]). The increase in muscle anabolism biomarkers was large after applying a single session (four studies; SMD = 1.29 [0.18; 2.41]) and moderate after applying a training programme (four studies; SMD = 0.58 [0.09; 1.06]). In conclusion, LL-BFRT increases muscle anabolism biomarkers to a greater extent than traditional LL training (low-quality evidence) or a passive control (moderate-quality evidence) in healthy older adults. This superior anabolic potential of LL-BFRT compared with LL training is sustained in the short to medium term. LL-BFRT is a safe training methodology for older adults, showing moderate-quality evidence of no increase in thrombotic biomarkers compared with traditional LL training.

## 1. Introduction

According to a recent report from the World Health Organisation, there are one billion individuals over 60 years of age, and this number is expected to double by 2050 [1]. Along with the increasingly sedentary behaviour of the older adult population, ageing contributes to progressive muscle impairment [2,3]. In fact, longitudinal studies have shown that muscle mass is lost at a rate of 0.64–0.70% and 0.80–00.98% per year in women and men aged 75 years, respectively [3]. The normal age-related loss of muscle mass should be carefully monitored because it can lead to sarcopenia if the loss occurs at an accelerated rate [4]. Sarcopenia should be considered of major importance in older adults, given that it is positively associated with increased risk of falls and fractures, disability, impairment in activities of daily living, poor quality of life, increased use of hospital services, institutionalization, and risk of all-cause mortality [4].

The gradual physiological loss of muscle mass in ageing is affected by genetic, physiological, and environmental factors and is the result of muscle protein breakdown (MPB) rates chronically exceeding muscle protein synthesis (MPS) rates [5]. Ageing is associated with a gradual decline in diverse growth factors (e.g., growth hormone [GH], insulin-like growth factor-1 [IGF-1]) [6] and shifts in myokine concentrations (e.g., myostatin, follistatin) [7], all of which promote MPB and, consequently, muscle wasting.

Exercise is a powerful non-pharmacological tool to delay ageing and has positive effects on older adult diseases [8]. Exercise training is highly desirable because it appears to enhance ageing-related hormone secretion, increasing GH [9], IGF-1 [9] and myokine concentrations [10], promoting MPS after exercise. In fact, aerobic training modalities [11,12] and resistance training protocols [13,14] achieve muscle gains in older adults. The intensity of training emerges as a pivotal factor, finding more pronounced neuromuscular and biochemical adaptations through high-load (HL) training in comparison with low-load (LL) training in older adults [15]. However, HL exercises might be contraindicated for older adults with specific pathological conditions [16,17]. LL blood flow restriction training (LL-BFRT) is a new training method currently being explored, showing promising results in older adults [18,19] who are often unable to exercise at HL [16]. LL-BFRT employs a pneumatic cuff to either completely or partially restrict arterial and venous blood flow while exercising [20]. The physiological stress attributable to LL-BFRT increases muscle mass and strength more than traditional LL training and to a similar extent when compared with HL training [18].

The effects of traditional exercise on muscle mass in older adults have recently been investigated from a biochemical standpoint, examining the behaviour of biomarkers related to muscle anabolism [21], but the biochemical changes involved in muscle mass gains in older persons who have undergone LL-BFRT have not yet been studied. Moreover, safety is a critical issue with LL-BFRT; although it is widely considered a safe training modality [22], complete vascular occlusion can trigger thrombus formation [23], which could be a significant risk for older adults who have high rates of thrombotic events [24]. Although there is evidence regarding the behaviour of biomarkers related to thrombus formation in older adults who undergo LL-BFRT, this evidence has not yet been pooled in a review. The main objective of this systematic review and meta-analysis is to determine the effects of LL-BFRT on muscle anabolism biomarkers and thrombotic biomarkers compared with the effects of traditional LL training and a passive control in healthy adults older than 60 years. As a secondary objective, we analysed the changes in these biomarkers in the short and medium term (acute/immediate and after at least 4 weeks of the training programme, respectively) in the same population.

## 2. Materials and Methods

This systematic review and meta-analysis adhered to the guidelines outlined by the Preferred Reporting Items for Systematic Reviews and Meta-Analyses (PRISMA) [25] (Prospero registration number: CRD42022364585).

### 2.1. Study Selection Criteria

The PICO (Population–Intervention–Comparison–Outcome of Interest–Study Design) strategy was employed to establish the clinical and methodological aspects concerning the inclusion or exclusion of the reviewed studies [26].

Population: The populations were healthy older adults without comorbidities who were over 60 years of age to meet the currently accepted senescence thresholds [27], with no gender limitation. None of those included had participated in scheduled training within the previous 3 months.

Intervention and comparison: Research should compare an LL-BFRT intervention with the following: (1) a traditional LL training intervention performed following the same protocol as LL-BFRT without the use of blood flow restriction and (2) a passive control group who did not perform a prescribed training protocol and continued their daily activities. Training interventions targeting the lower and/or upper limbs were included.

Outcomes: Research should include muscle anabolism biomarkers and thrombosis biomarkers. IGF-1 [28], GH [28], N-terminal procollagen type III peptide (P3NP) [29], C-terminal Agrin (CAF) [29], myostatin [30], and follistatin [30] were considered biomarkers of muscle anabolism. In terms of safety, fibrin/fibrinogen degradation products (FDP), D-dimer, C-reactive protein (CRP) and thrombomodulin were considered thrombotic biomarkers based on a recent consensus [31].

Study design: Only randomized controlled trials (RCTs) and cross-over trials were considered. 

### 2.2. Search Strategy

Russell-Rose et al.’s [32] recommendations were followed to design the search strategy. The following electronic databases were searched until 1 March 2024: MEDLINE, Web of Science, PEDro, Scopus, CINHAL, Science Direct, and CENTRAL. Data in Scheme S1 shows how the search string was designed for each database. In addition, references from preceding systematic reviews within this domain were consulted. When supplementary details were necessary from the studies, the authors were contacted via email. The search was conducted by two independent reviewers utilizing the same criteria (RFG and MGV). A third reviewer (ILUV) participated in resolving disagreements.

### 2.3. Selection Criteria and Data Extraction

In the initial stage, evaluators (RFG and MGV) scrutinized study titles, abstracts, and keywords in accordance with the Cochrane guidelines [33]. The subsequent phase entailed a comprehensive evaluation of complete texts to ensure adherence to the selection criteria, documenting reasons for exclusion. Discrepancies underwent resolution through consultation with a third reviewer (ILUV). Data extraction was conducted by 2 reviewers (RFG and MGV).

### 2.4. Methodological Quality and Risk-of-Bias Assessment

The PEDro scale was utilized to assess the methodological quality of the incorporated trials, which is a reliable tool for evaluating RCTs [34]. Comprising 11 items with a maximum score of 10 points, the PEDro scale stratified the total score for each study into the following categories: poor (<4 points), fair (4–5 points), good (6–8 points) and excellent (9–10 points) [35]. Following the Cochrane guidelines [33], the risk of bias for each included study was evaluated. Each criterion’s risk of bias was categorized as high, low, or uncertain, with recorded justifications. The “other biases” criterion was elaborated to specify items that could potentially bias the results.

The quality and risk of bias assessments were conducted by two independent, trained reviewers (RFG and MGV) employing identical methodologies. In cases of disagreement, a third reviewer (ILUV) was consulted. Inter-rater reliability was assessed utilizing the Kappa coefficient, where interpretations of values were as follows: (1) >0.81–1.00 excellent agreement among the evaluators; (2) 0.61–0.80 good agreement; (3) 0.41–0.60 moderate agreement; and (4) 0.21–0.40 poor agreement [36].

### 2.5. Qualitative Analysis

The qualitative analysis followed the Grading of Recommendations, Assessment, Development, and Evaluation (GRADE) framework, in accordance with the guidelines proposed by Andrews et al. [37].

### 2.6. Data Analysis

The statistical analysis was performed using RStudio 3.0 software, employing the “meta” and “esc” packages. All significance tests were conducted at a 5% level. Meta-analysis was carried out solely if there were data present from at least 3 RCTs.

To improve the precision and extend the generalizability of our analyses, multiple trials from diverse studies (e.g., GH and IGF-1) were included in all analyses. Mean difference and standard deviation (SD) values for muscle anabolism and thrombotic risk were employed for both pre- and post-intervention analyses to compute the standardized mean difference (SMD). The change in SD was computed in accordance with the Cochrane recommendations [33]. If needed, mean scores and SD were estimated from graphs. In the comparison between LL-BFRT and LL training for muscle anabolism biomarkers, the data synthesis was categorized by group according to when the measurement was taken as follows: (1) within 30 min after a single training session and (2) after a programme of at least 6 weeks of training. In the event that a study had several measurements of the same variable within 30 min after the training session, the pooled mean and SD were calculated for use in the analysis.

Forest plots were utilized to present summary statistics for all analyses. A random-effects model was employed to determine the overall effect size (standardized mean differences, SMDs). Hedges’ g was used to examine the effect size of the statistical significance of the overall SMD, with interpretation as follows: (1) trivial effect (g < 0.20); (2) small effect (g = 0.20–0.49); (3) moderate effect (g = 0.50–0.79); and (4) large effect (g ≥ 0.80). Confidence intervals around the pooled effect were obtained using Knapp–Hartung adjustments [38].

Heterogeneity within the studies was assessed utilizing Cochran’s Q statistic test and the inconsistency index (I^2^) [39]. Heterogeneity was deemed present if Cochran’s Q statistic test yielded significance (*p* < 0.1) and/or if I^2^ exceeded 50% [40]. To facilitate clinical interpretation and offer more insightful inferences, the prediction interval based on the between-study variance tau-squared (τ^2^) was provided. This prediction interval estimates the genuine intervention effect anticipated in future settings [41]. For continuous outcomes, the restricted maximum likelihood estimator was utilized to calculate the between-study variance τ^2^, as recommended for continuous outcomes [42].

An exclusion sensitivity analysis was conducted to evaluate the potential impact of studies on the results of the meta-analysis and to assess its robustness. Publication bias was investigated through visual inspection of funnel plots, where an asymmetric plot suggested potential bias. The Luis Furuya-Kanamori (LFK) index, recognized for its sensitivity in detecting publication bias in meta-analyses with a limited number of studies, was utilized as a quantitative measure [43]. Interpretations of LFK values were as follows: (1) no asymmetry (LFK within ±1); (2) minor asymmetry (LFK exceeding ±1 but within ±2); and (3) major asymmetry (LFK exceeding ±2). In the case of significant asymmetry, the Duval and Tweedie trim and fill method, a small-study effect correction technique, was applied to address potential publication bias [44].

## 3. Results

### 3.1. Study Selection

**Figure 1:**
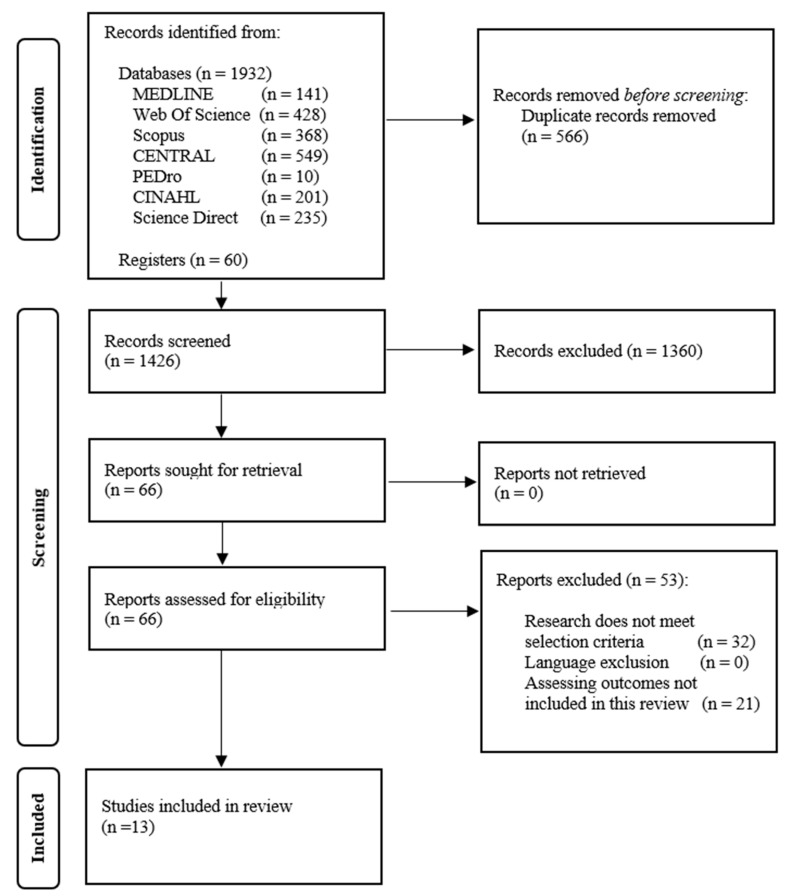
PRISMA flow diagram.

A total of 1992 records were yielded through the database and cross-reference search. After removing duplicate records and after screening study titles and abstracts, 66 articles were full-text reviewed. Finally, 13 articles met the selection criteria and were included in the final analysis. Figure 1 illustrates a flowchart detailing the search strategy.

### 3.2. Characteristics of the Included Studies

The studies aggregated 256 healthy older adults (mean (min–max) age 68 (62–71) years, 44.53% female) (Table 1). Ten studies evaluated the effects of LL-BFRT versus traditional LL training [45,46,47,48,49,50,51,52,53,54], and six studies assessed the effects of LL-BFRT versus a passive control [46,49,52,55,56,57]. Only 2 studies performed an aerobic LL-BFRT (45% heart rate reserve) [46,54], and 11 studies performed a resistance LL-BFRT (20–45% one-repetition maximum) [45,47,48,49,50,51,52,53,55,56,57]. The employed cuff pressures varied widely (59 mmHg [49,52] to 270 mm Hg [48,50,56]), while cuff widths ranged from 3 cm [48,50] to 13 cm [51], with 5 cm being the most widely used [46,49,52,54,55,56]. Of the nine studies that evaluated biomarkers related to muscle anabolism [45,46,47,49,51,52,53,54,57], four evaluated GH [45,47,51,54], four evaluated IGF-1 [45,51,53,57], two evaluated CAF and P3NP [46,49], and one evaluated myostatin and follistatin [52] concentrations. Among the studies that evaluated anabolic biomarkers, four assessed them in the short term (≤30 min post-training session) [45,47,51,54], and four assessed them in the medium term (≥6 weeks, measurements performed at least 48 h after the last training session) [46,49,52,53]. Of the six studies that evaluated biomarkers related to thrombotic risk [47,48,50,53,55,56], four evaluated FDP and D-dimer [48,50,55,56], one evaluated CRP [53], and one evaluated thrombomodulin [47] (Table 1). All the studies that evaluated thrombotic risk were conducted in the medium term (≥4 weeks, measurements performed at least 24 h after the last training session) [47,48,50,53,55,56]. The training programmes lasted between 4 weeks [47] and 12 weeks [48,50,53,55,56], exercising between two times [48,50,55,56] and three times [46,47,49,52,53,57] per week. Only three studies used one training session [45,51,54]. Seven studies performed lower limb training [45,46,51,54,55,56,57], two studies performed upper limb training [48,50], and four studies performed upper and lower limb training [47,49,52,53] (Table 1). All included studies reported no adverse events, but only one of them explicitly stated the absence of adverse events [53].

### 3.3. Methodological Quality and Risk of Bias of the Included Studies

The mean PEDro score for the studies included was 5.9, with scores ranging from 5 to 7 (Table 2). Inter-rater reliability exhibited a high level of agreement among assessors (k = 0.87).

The risk of bias assessment summary for the trials included is depicted in Figure S1. The overall risk of bias in the trials included in the present meta-analysis was elevated. The most substantial risk of bias was observed in adequate stopping rules and conflicts of interest, with at least five studies categorized as having a high risk of bias. However, more than 65% had an unclear risk of randomisation concealment and selective reporting (no study had a low risk of bias on the latter criterion). All evaluated studies strictly adhered to well-defined protocols concerning their implementation, which is a critical necessity in exercise interventions to mitigate the risk of differential behaviour by personnel administering the intervention [58]. In addition, all groups were deemed to have a low risk of bias in blinding participants and assessors, given the objective and challenging-to-bias nature of blood marker measurements.

### 3.4. Muscle Anabolism Biomarkers

**Figure 2:**
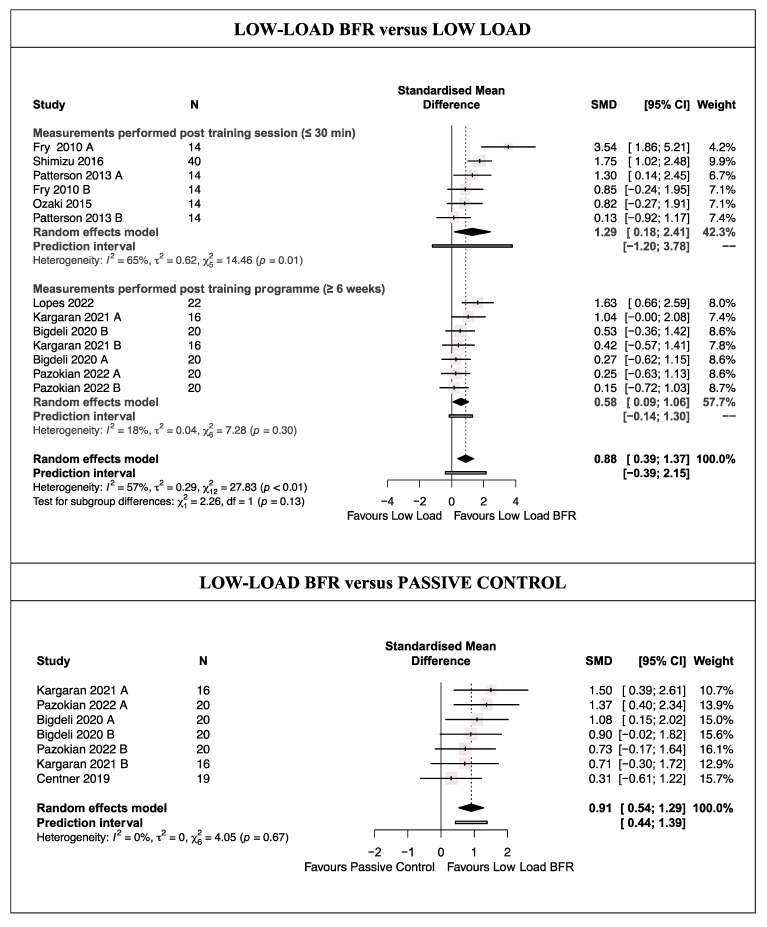
Synthesis Forest plot for muscle anabolism [45,46,47,49,51,52,53,54,57].

There was low-quality evidence from eight studies [45,46,47,49,51,52,53,54] (13 trials; *n* = 160) that LL-BFRT produces a large and statistically significant increase in muscle anabolism biomarkers compared with traditional LL training (SMD = 0.88 [0.39; 1.37]; Figure 2 and Table 3). Heterogeneity was significant (Q = 27.83 [*p* < 0.01]; I^2^ = 57%), and the prediction interval crossed zero (−0.39; 2.15); future studies might therefore find conflicting results. No single study significantly affected the overall SMD; however, evidence of publication bias was detected (asymmetric funnel plot shape; minor asymmetry (LFK index = 1.54)) (Figure S2). When the sensitivity analysis was adjusted for publication bias, there was no influence on the estimated effect because the trim and fill method considered that no studies should be added. Therefore, the initial results were maintained. The meta-analysis results for the subgroups showed no statistically significant difference in muscle anabolism biomarkers between the overall SMD obtained after a single session or a more than 6-week-long programme of LL-BFRT compared with traditional LL training (*p* = 0.13) (Figure 2). However, the increase in muscle anabolism biomarkers was large after applying a single session (low-quality evidence; four studies [45,47,51,54] and six trials; *n* = 82; SMD = 1.29 [0.18; 2.41]), whereas it was moderate after applying a training programme (moderate-quality evidence; four studies [46,49,52,53] and seven trials; *n* = 78; SMD = 0.58 [0.09; 1.06]).

There was moderate-quality evidence from four studies [46,49,52,57] (seven trials; *n* = 75) that LL-BFRT produces a large and statistically significant increase in muscle anabolism biomarkers compared with a passive control without training (SMD = 0.91 [0.54; 1.29]; Figure 2 and Table 3). Heterogeneity was not significant (Q = 4.05 [*p* = 0.67]; I^2^ = 0%), and the prediction interval did not cross zero (0.44; 1.39), making the observed results more robust. No single study significantly affected the overall SMD, and no evidence of publication bias was detected (symmetric funnel plot shape; no asymmetry (LFK index = 0.47) (Figure S2).

### 3.5. Thrombotic Biomarkers

**Figure 3:**
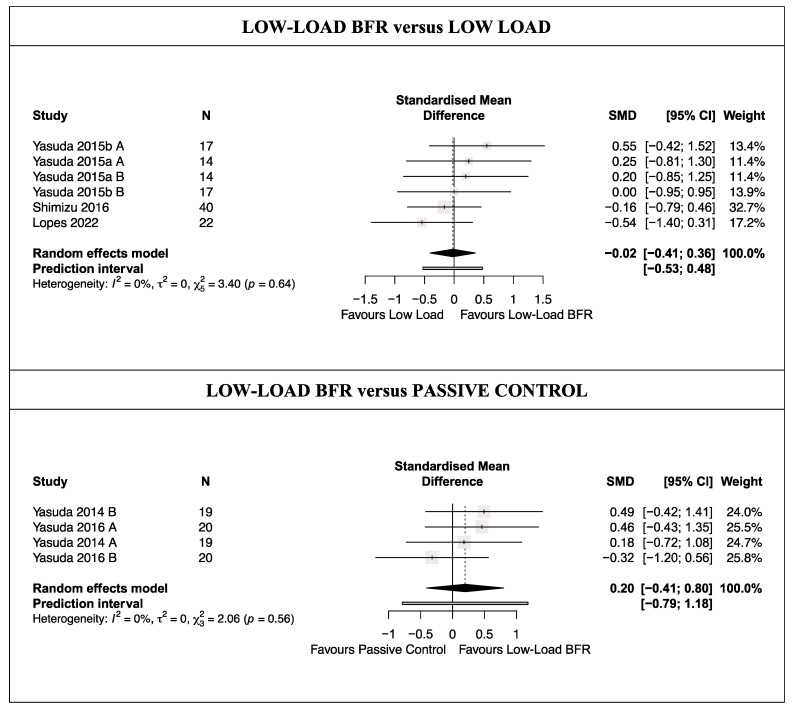
Synthesis forest plot for thrombotic risk [47,48,50,53,55,56].

There was moderate-quality evidence from four studies [47,48,50,53] (six trials; *n* = 93) that LL-BFRT does not produce a statistically significant increase in thrombotic biomarkers compared with traditional LL training (SMD = −0.02 [−0.41; 0.36]; Figure 3 and Table 3). Heterogeneity was not significant (Q = 3.40 [*p* = 0.64]; I^2^ = 0%). No single study significantly affected the overall SMD, and no evidence of publication bias was detected (symmetric funnel plot shape; no asymmetry [LFK index = 0.83]; Figure S3).

There was low-quality evidence from two studies [55,56] (four trials; *n* = 39) that LL-BFRT does not produce a statistically significant increase in thrombotic biomarkers compared with a passive control (SMD = 0.20 [−0.41; 0.80]; Figure 3 and Table 3). Heterogeneity was not significant (Q = 2.06 [*p* = 0.56]; I^2^ = 0%). No single study significantly affected the overall SMD, and no evidence of publication bias was detected (symmetric funnel plot shape; no asymmetry (LFK index = 0.21)) (Figure S3).

## 4. Discussion

The present systematic review and meta-analysis quantified, for the first time, the effect of LL-BFRT compared with traditional LL training or a passive control regarding muscle anabolism and thrombotic biomarkers in healthy adults older than 60 years. The results revealed that, based on the biochemical environment, LL-BFRT had a larger potential to increase muscle anabolism compared with traditional LL training or non-exercise. However, these results were only robust with respect to the comparison of LL-BFRT with passive control when the prediction interval was taken into account. Changes in muscle anabolism biomarkers were comparable at the end of a single training session and at the end of a ≥6-week training period, suggesting that the anabolic potential is sustained throughout the training period. Additionally, LL-BFRT did not seem to elevate thrombotic biomarkers more than traditional LL training or non-exercise, suggesting that LL-BFRT is a safe training method even for older adults.

It has been widely documented that exercise produces functional benefits and muscle structural adaptations in older adults [8,13]. It is therefore not surprising that LL-BFRT generates a propitious biochemical environment for muscle anabolic adaptations. Mechanical loading in conjunction with growth factors extensively evaluated by the included studies in this review (e.g., GH and IGF-1) are the most potent stimuli for increasing MPS, share similar signalling pathways, and are only present during exercise [59,60]. Mechanical loading and growth factors are transduced into intracellular signals that converge in the phosphorylation of the TSC2 [59], disinhibition of rheb [59], and subsequent activation of the mammalian target of rapamycin complex 1 (mTORC1), which is considered one of the main protein complexes involved in MPS regulation [59,60]. Further increases in GH and IGF-1 concentrations, as found in this review, as well as elevated levels of mTORC-1 activation in parallel, have been reported after performing LL-BFRT in older adults [45].

Several reviews have highlighted the higher hypertrophic potential of LL-BFRT compared with traditional LL training in older adults [18,19]. The current systematic review shows that LL-BFRT presents a greater capacity for modifying muscle anabolism biomarkers, providing further insight into LL-BFRT-induced muscle hypertrophy in older adults. LL-BFRT-associated hypoxia likely plays a key role in these adaptations. Local muscle hypoxia enhances glycolytic cell metabolism and lactate accumulation [45,47,54], influencing the biochemical environment through various pathways. It has been documented that the acidic intramuscular environment can stimulate the secretion of growth factors such as GH [61]. Increased GH concentrations can then stimulate hepatic IGF-1 synthesis and release (according to our knowledge) into the GH/IGF-1 axis [62]. However, recent studies have suggested that GH is unlikely to stimulate IGF-1 secretion in such a brief period [63]. The mechanisms underlying the increase in IGF-1 levels following LL-BFRT therefore need further examination. Based on empirical evidence, Loenneke et al. [64] suggested that metabolite accumulation and hypoxic stimulus reduce the concentrations of myostatin, a negative regulator of MPS [30]. Localised muscle cell swelling is generated by metabolite accumulation and the pressure conditions of LL-BFRT [65], which is associated with IGF-1, GH, and testosterone secretion [65]. CAF concentrations decrease after LL-BFRT in older adults [46,49], suggesting less neuromuscular junction degradation [29], a process that could involve the activation of group III and IV afferent fibres due to metabolite accumulation, leading to fast-twitch muscle fibre recruitment [64]. Thus, the neuromuscular activation with LL-BFRT is higher than with LL training, protecting the neuromuscular junction to a greater extent against ageing degeneration [66]. All these mechanisms occur because of the cuff-imposed blood restriction, which might explain the greater changes detected in the biochemical environment with LL-BFRT than with traditional LL training.

Previous studies have suggested that increases in GH and IGF-1 concentrations after traditional LL resistance training are insufficient to induce MPS [60]. However, the results of this review attribute the greater secretion of growth factors to LL-BFRT, allowing a biochemical environment that could increase MPS more than previously studied with traditional LL training. Several mechanisms could be involved in the increase in MPS due to growth factors secreted with LL-BFRT. First, growth factors activate mTORC1 signalling, as already reported in previous clinical trials [45,67]. Second, increases in IGF-1 concentrations induce satellite cell proliferation and differentiation [68] and reduce autophagy-mediated MPB through the ubiquitin–proteasome system [68]. IGF-1 could neutralise the interleukin-6 muscular catabolic effect [69]; however, conflicting results in the literature [70] suggest that the anti-inflammatory capacity of LL-BFRT should be further studied. Lastly, Basin et al. [71] demonstrated that higher concentrations of IGF-1 and GH induce increases in P3NP concentrations, suggesting that muscular structural remodelling is in progress [29]. As a whole, there is support from the physiological standpoint to justify the superior anabolic potential and increases in muscle mass and strength detected with LL-BFRT compared with LL training or non-exercise [18,19].

This review showed no difference between the concentration of biomarkers of muscle anabolism after a single session or a 6-week or longer programme of LL-BFR compared with traditional LL training. Based on our results, the anabolic potential of LL-BFRT appears to be the same in the short and medium term, suggesting no tolerance to the anabolic stimuli induced by training. Anabolic resistance is a process that diminishes the ability to increase MPS and adapt to anabolic stimulation, which occurs frequently with ageing [7]. The literature indicates that LL-BFRT can induce muscle anabolic changes by increasing the MPS/MPB ratio in older adults [45,65]. This anabolic response is mediated primarily by those biomarkers of muscle anabolism that this review detected to be elevated even up to the medium term. We therefore hypothesise that LL-BFRT can attenuate anabolic resistance in older adults.

This systematic review concluded that LL-BFRT does not modify the concentrations of thrombotic biomarkers more than traditional LL training or a passive control in older adults. These results are consistent with other studies that have stated that individuals exposed to LL-BFRT were no more likely to experience adverse events than those exposed to exercise alone [72]. LL-BFRT was previously considered a safe training methodology from a qualitative standpoint [22,23], but this review now supports this statement quantitatively. The survey by Nakajima et al. [73] supports these results, which included more than 12,000 patients of all age groups who underwent LL-BFRT sessions, showing that the incidence of venous thrombus and pulmonary embolism was as low as 0.055% and 0.008%, respectively. Other trials have detected an increase in fibrinolytic capacity after LL-BFRT in healthy participants, further corroborating the safety of LL-BFRT [74].

The present review provides physiological support for the increase in the concentration of biomarkers of muscle anabolism after LL-BFRT and the strongest evidence to date in confirming the safety of LL-BFRT for the older adult population exclusively, with low rates of thrombotic events. In clinical practice, LL-BFRT could be an interesting option for older adults who are contraindicated with HL training to obtain similar results in terms of strength, mass, and functional capacity.

There are several limitations in this systematic review and meta-analysis, which should be noted. The small sample size and the high risk of bias in the included trials indicate that the interpretation of the results should be performed cautiously, particularly given that some trials were conducted by the same author groups. The included studies present significant methodological differences in terms of determining cuff pressure, cuff width, duration of training programmes, trained limbs, and exercise prescription. This heterogeneity prevents us from discerning the actual effect of LL-BFRT. In addition, the long-term behaviour of muscle anabolism biomarkers cannot yet be studied due to a lack of studies.

## 5. Conclusions

This meta-analysis shows that LL-BFRT increases muscle anabolism biomarkers to a greater extent than traditional LL training (low-quality evidence) or a passive control (moderate-quality evidence) in healthy adults older than 60 years of age, which could lead to significant muscle structural adaptations. This superior anabolic potential of LL-BFRT compared with LL training is sustained in the short (low-quality evidence) to medium term (moderate-quality evidence). However, the superiority of muscle anabolism biomarkers of LL-BFRT versus LL training should be interpreted with caution according to the prediction intervals, given that the inclusion of new studies could modify the results. This review also provides preliminary evidence that LL-BFRT is a safe training methodology even for older adults, showing no increase in thrombotic biomarkers compared with traditional LL training (moderate-quality evidence). However, the high risk of bias and poor reporting quality in the reviewed studies precludes firm conclusions. Future clinical studies need to follow higher standards of methodological quality and reporting to advance this promising field.

## Figures and Tables

**Table 1:** Methodological characteristics and results of the included studies.

Study	Participants	Intervention Groups	Training Protocol	Outcomes	Key Results
**Bigdeli et al., 2020** [49] RCT	*N* = 30 (30 males) Age: 68 ± 6 years	LL-BFRT (25–35% 1RM) (*N* = 10) Pressure: Upper limb: ∼59–82 mmHg Lower limb: ∼115–161 mmHg Cuff: 5 cm width (Ghamat pooyan, Tehran, Iran) LL training (25–35% 1RM) (*N* = 10) Passive control (*N* = 10)	Exercise mode: 11 functional upper and lower limb exercises Volume: 2–4 × 10 set × reps Frequency: 6 wk; 3 days/wk	Biomarkers of muscle anabolism: CAF (Pre–post 6 wk [48 h from last session]). P3NP (Pre–post 6 wk [48 h from last session]).	Biomarkers of muscle anabolism: LL-BFRT showed significantly higher CAF levels than the control passive at 6 wk. However, there were no significant differences between LL-BFRT and LL training with respect to CAF and P3NP levels between the intervention groups.
**Centner et al., 2019** [57] RCT	*N* = 19 (19 males) Age: 62 ± 8 years	LL-BFRT (20–30% 1RM) (*N* = 11) Pressure: 113.2 ± 19.5 mmHg Cuff: 12 cm (Zimmer Biomet, Warsaw, IN, USA) Passive control (*N* = 8)	Exercise mode: Leg press Volume: 4 × 15–30 set × reps Frequency: 8 wk; 3 days/wk	Biomarkers of muscle anabolism: IGF-1 (Pre–post 8 wk [72–168 h from last session]).	Biomarkers of muscle anabolism: No significant differences between groups were found in IGF-1 concentrations at 8 wk.
**Fry et al., 2010** [45] Crossover trial	*N* = 7 (7 males) Age: 70 ± 2 years	LL-BFRT (20% 1RM) (*N* = 7) Pressure: 200 mmHg Cuff: Width not reported (Kaatsu-Master Mini, Sato Sports Plaza, Tokyo, Japan) LL training (20% 1RM) (*N* = 7)	Exercise mode: Bilateral leg extension Volume: 4 × 15–30 set × reps Frequency: 1 session per protocol	Biomarkers of muscle anabolism: GH (Pre, 15–30–45–60–90–120–150–180 min post 1 session). IGF-1 (Pre–1 h–3 h post 1 session). Biomarkers of thrombotic risk: D-dimer (Pre–15 min post 1 session).	Biomarkers of muscle anabolism: LL-BFRT showed significant increases in GH concentration compared with LL training at 15 and 30 min after the end of exercise. There were no significant differences between groups with respect to IGF-1 levels, although LL training significantly reduced its concentrations 3 h after the session.Biomarkers of thrombotic risk: No significant differences between groups were found in D-dimer concentrations in any of the assessments.
**Kargaran et al., 2021** [46] RCT	*N* = 24 (24 females) Age: 63 ± 3 years	LL-BFRT (45% HRR) (*N* = 8) Pressure: 150–200 mmHg Cuff: 5 cm (Ghamat pooyan, Tehran, Iran) LL training (45% HRR) (*N* = 8) Passive control (*N* = 8)	Exercise mode: Walking on a treadmill Volume: 20 min Frequency: 8 wk; 3 days/wk	Biomarkers of muscle anabolism: P3NP (Pre–post 8 wk [48 h from last session]). CAF (Pre–post 8 wk [48 h from last session]).	Biomarkers of muscle anabolism: There was significant reductions in CAF levels in the LL-BFRT group compared with the two controls at 8 wk. there was only a significant increased in P3NP in the LL-BFRT group compared with the passive control at 8 wk.
**Lopes et al., 2022** [53] RCT	*N* = 22 (14 females/8 males) Age: 71 ± 7 years	LL-BFRT (30% 1RM) (*N* = 12) Pressure: 65 ± 5 mmHg Cuff: 11 × 85 cm (Hokanson 105 model TD312, Bellevue, WA, USA) LL training (30% 1RM) (*N* = 10)	Exercise mode: Elbow flexion/extension, leg press and knee extension Volume: 3 × 10 set × reps Frequency: 12 wk; 3 days/wk	Biomarkers of muscle anabolism: IGF-1 (Pre–post 12 wk [48 h from last session]). Biomarkers of thrombotic risk: CRP (Pre–post 12 wk [48 h from last session]).	Biomarkers of muscle anabolism: LL-BFRT showed significant increases in IGF-1 concentration compared with LL training at 12 wk.Biomarkers of thrombotic risk: No significant differences between groups were found in CRP concentrations at 12 wk.
**Ozaki et al., 2015** [54] Crossover trial	*N* = 7 (7 females) Age: 64 ± 2 years	LL-BFRT (45% HRR) (*N* = 7) Pressure: 180–200 mmHg Cuff: 5 cm width [54] LL training (45% HRR) (*N* = 7)	Exercise mode: Walking on a treadmill Volume: 20 min Frequency: 1 session per protocol	Biomarkers of muscle anabolism: GH (Pre–post-15 min post 1 session).	Biomarkers of muscle anabolism: No significant differences between groups were found in GH concentrations in any of the assessments.
**Patterson et al., 2013** [51] Crossover trial	*N* = 7 (7 males) Age: 71 ± 6 years	LL-BFRT (20% 1RM) (*N* = 7) Pressure: 110 mmHg Cuff: 13 cm width (D.E. Hokanson, Inc., Bellevue, WA, USA) LL training (20% 1RM) (*N* = 7)	Exercise mode: Knee extension Volume: 5 × failure set × reps Frequency: 1 session per protocol	Biomarkers of muscle anabolism: GH (Pre-30–60–120 min post 1 session). IGF-1 (Pre-30–60–120 min post 1 session).	Biomarkers of muscle anabolism: LL-BFRT showed significant increases in GH concentration at 30 min post-session compared with LL training. There were no significant differences between groups in IGF-1 concentrations in any of the assessments.
**Pazokian et al., 2022** [52] RCT	*N* = 30 (30 males) Age: 68 ± 6 years	LL-BFRT (25–35% 1RM) (*N* = 10) Pressure: Upper limb: ∼59–82 mmHg Lower limb: ∼115–161 mmHg Cuff: 5 cm width (Ghamat pooyan, Tehran, Iran) LL training (25–35% 1RM) (*N* = 10) Passive control (*N* = 10)	Exercise mode: 11 functional upper and lower limb exercises Volume: 2–4 × 10 set × reps Frequency: 6 wk; 3 days/wk	Biomarkers of muscle anabolism: Myostatin (Pre–post 6 wk [48 h from last session]). Follistatin (Pre–post 6 wk [48 h from last session]).	Biomarkers of muscle anabolism: LL-BFRT and LL training showed significant increases in follistatin and decreases in myostatin concentration compared with the passive control at 6 wk. No differences were found between LL-BFRT and LL training on those substances at 6 wk.
**Shimizu et al., 2016** [47] RCT	*N* = 40 (33 males/7 females) Age: 71 ± 4 years	LL-BFRT (20% 1RM) (*N* = 20) Pressure: 134 ± 16 mmHg upper limb; 163 ± 17 lower limb Cuff: 10 and 7 cm width, lower and upper limb respectively LL training (20% 1RM) (*N* = 20)	Exercise mode: Leg extension, leg press, rowing and chest press Volume: 3 × 20 set × reps Frequency: 4 wk; 3 days/wk	Biomarkers of muscle anabolism: GH (Pre–post 1 session) Biomarkers of thrombotic risk: Thrombomodulin (Pre–post 4 wk [24 h from last session]).	Biomarkers of muscle anabolism: LL-BFRT obtained significant increases in GH levels compared with LL training after the first training session.Biomarkers of thrombotic risk: No significant differences between groups were found in thrombomodulin concentrations at 4 wk.
**Yasuda et al., 2014** [56] RCT	*N* = 19 (5 males/14 females) Age: 69 ± 7 years	LL-BFRT (20–30% 1RM) (*N* = 9) Pressure: 120–270 mmHg Cuff: 5 cm width (KAATSU Master, Sato Sports Plaza, Tokyo, Japan) Passive control (*N* = 10)	Exercise mode: Knee extension and leg press Volume: 4 × 10–30 set × reps Frequency: 12 wk; 2 days/wk	Biomarkers of thrombotic risk: FDP (Pre–post 12 wk [72–168 h from last session]). D-dimer (Pre–post 12 wk [72–168 h from last session]).	Biomarkers of thrombotic risk: No significant differences were found between groups in FDP and D-dimer concentrations at 12 wk.
**Yasuda et al., 2015a** [48] RCT	*N* = 14 (14 females) Age: 69 ± 7 years	LL-BFRT (26–30% 1RM) (*N* = 7) Pressure: 120–270 mmHg Cuff: 3 cm width (KAATSU Master, KAATSU Japan Co., Ltd., Tokyo, Japan) LL training (28–30% 1RM) (*N* = 7)	Exercise mode: Arm curl and triceps press down with elastic bands Volume: 4 × 15–30 set × reps Frequency: 12 wk; 2 days/wk	Biomarkers of thrombotic risk: FDP (Pre–post 12 wk [72–168 h from last session]). D-dimer (Pre–post 12 wk [72–168 h from last session]).	Biomarkers of thrombotic risk: No significant differences were found between groups in FDP and D-dimer concentrations at 12 wk.
**Yasuda et al., 2015b** [50] RCT	*N* = 17 (3 males/14 females) Age: 70 ± 6 years	LL-BFRT (826–30% 1RM) (*N* = 9) Pressure: 120–270 mmHg Cuff: 3 cm width (KAATSU Master, KAATSU Japan Co., Ltd., Tokyo, Japan) LL training (28–30% 1RM) (*N* = 8)	Exercise mode: Arm curl and triceps press down with elastic bands Volume: 4 × 15–30 set × reps Frequency: 12 wk; 2 days/wk	Biomarkers of thrombotic risk: FDP (Pre–post 12 wk [72–168 h from last session]). D-dimer (Pre–post 12 wk [72–168 h from last session]).	Biomarkers of thrombotic risk: No significant differences were found between groups in FDP and D-dimer concentrations at 12 wk.
**Yasuda et al., 2016** [55] RCT	*N* = 20 (20 females) Age: 69 ± 6 years	LL-BFRT (35–45% 1RM) (*N* = 10) Pressure: 120–200 mmHg Cuff: 5 cm width (KAATSU Master, KAATSU Japan Co., Ltd., Tokyo, Japan) Passive control (*N* = 10)	Exercise mode: Squat and knee extension with elastic bands Volume: 3 × 15–30 set × reps Frequency: 12 wk; 2 days/wk	Biomarkers of thrombotic risk: FDP (Pre–post 12 wk [72–168 h from last session]). D-dimer (Pre–post 12 wk [72–168 h from last session]).	Biomarkers of thrombotic risk: No significant differences were found between groups in FDP and D-dimer concentrations at 12 wk.

1RM: one repetition maximum; CAF: C-terminal Agrin Fragment; CRP: C-reactive protein; CI: confidence interval; FDPs: fibrin/fibrinogen degradation products; GH: growth hormone; HRR: heart rate reserve; IGF-1: insulin-like growth factor I; LL: low load; LL-BFR: low-load blood flow restriction; P3NP: *Procollagen III N-terminal peptide*; RCT: randomized controlled trial; Rep: repetitions; Wk: week.

**Table 2:** PEDro scores for the included studies (*n* = 13).

Study	Random Allocation	Concealed Allocation	Groups Similar at Baseline	Participant Blinding	Therapist Blinding	Assessor Blinding	<15% Dropouts	Intention- to-Treat Analysis	Between-Group Difference Reported	Point Estimate and Variability Reported	TOTAL
**Bigdeli 2020** [49]	Y	N	Y	N	N	Y	Y	Y	Y	Y	**7**
**Centner 2019** [57]	Y	Y	Y	N	N	Y	N	N	Y	Y	**6**
**Fry 2010** [45]	Y	N	Y	N	N	N	Y	Y	Y	Y	**6**
**Kargaran 2021** [46]	Y	N	Y	N	N	Y	Y	Y	Y	Y	**7**
**Lopes 2022** [53]	Y	N	Y	N	N	N	Y	N	Y	Y	**5**
**Ozaki 2015** [54]	Y	N	Y	N	N	N	Y	Y	Y	Y	**6**
**Patterson 2013** [51]	N	N	Y	N	N	N	Y	Y	Y	Y	**5**
**Pazokian 2013** [52]	Y	N	Y	N	N	N	Y	Y	Y	Y	**6**
**Shimizu 2016** [47]	Y	N	Y	N	N	N	Y	Y	Y	Y	**6**
**Yasuda 2016** [55]	Y	N	Y	N	N	N	Y	Y	Y	Y	**6**
**Yasuda 2015a** [48]	Y	N	Y	N	N	N	Y	Y	Y	Y	**6**
**Yasuda 2015b** [50]	Y	N	Y	N	N	N	Y	Y	Y	Y	**6**
**Yasuda 2014** [56]	Y	N	Y	N	N	N	Y	N	Y	Y	**5**
N = No, Y = Yes								**Mean**			**5.9**

**Table 3:** GRADE evidence profile for the effects of low-load blood flow restriction training.

Outcome Comparison; Number of Studies (Trials); Sample Size	Risk of Bias	Inconsistency	Indirectness of Evidence	Imprecision	Publication Bias	SMD (95% CI)	Certainty of Evidence
**Muscle Anabolism**							
LL-BFR vs. LL (overall effect); eight studies (13 trials); *n* = 160 Single training session: four studies (six trials); *n* = 82 Programme training: four studies (seven trials); *n* = 78	Not serious Not serious Not serious	Serious ^b^ Serious ^b^ Not serious	Not serious Not serious Not serious	Serious ^c^ Serious ^c^ Serious ^c^	Not serious Not serious Not serious	0.88 (0.39 to 1.37) * 1.29 (0.18 to 2.41) * 0.58 (0.09 to 1.06) *	⨁⨁ LOW ⨁⨁ LOW ⨁⨁⨁ MODERATE
LL-BFR vs. passive control (overall effect): four studies (seven trials); *n* = 75	Not serious	Not serious	Not serious	Serious ^c^	Not serious	0.91 (0.54 to 1.29) *	⨁⨁⨁ MODERATE
**Thrombotic risk**							
LL-BFR vs. LL (overall effect): four studies (six trials); *n* = 93	Not serious	Not serious	Not serious	Serious ^c^	Not serious	–0.02 (–0.41 to 0.36)	⨁⨁⨁ MODERATE
LL-BFR vs. passive control (overall effect): two studies (four trials); *n* = 39	Serious ^a^	Not serious	Not serious	Serious ^c^	Not serious	0.20 (–0.41 to 0.80)	⨁⨁ LOW

* Statistically significant differences; ^a^ more than 50% of the studies/trials presented the risk of bias for attrition; ^b^ I^2^ > 50%; ^c^ sample size less than 400 patients. LL-BFR, low-load blood flow restriction; LL, low load. Levels of evidence: ⨁⨁ low; ⨁⨁⨁ moderate.

## Data Availability

No new data were created.

## Supplementary Materials

The following supporting information can be downloaded at: https://www.mdpi.com/article/10.3390/life14030411/s1, Scheme S1. Full search strategy; Figure S1. Risk of bias summary and graph; Figure S2. Sensitivity and publication bias funnel plots for the comparison in muscle anabolism; Figure S3. Sensitivity and publication bias funnel plots for the comparison in thrombotic risk.
