# Supplementary material for: Effects of Low-Load Blood Flow Restriction Training on Muscle Anabolism Biomarkers and Thrombotic Biomarkers Compared with Traditional Training in Healthy Adults Older Than 60 Years: Systematic Review and Meta-Analysis
Source: Life (Basel). 2024 Mar 20;14(3):411. doi: 10.3390/life14030411 (PMC10971244; doi:10.3390/life14030411)
Supplement: Supplementary file 1 [file life-14-00411-s001.zip › life-2866246 supplementary/Figure S1. Risk of bias summary and graph final ok.pdf]

Figure S1. Risk of bias summary and graph.

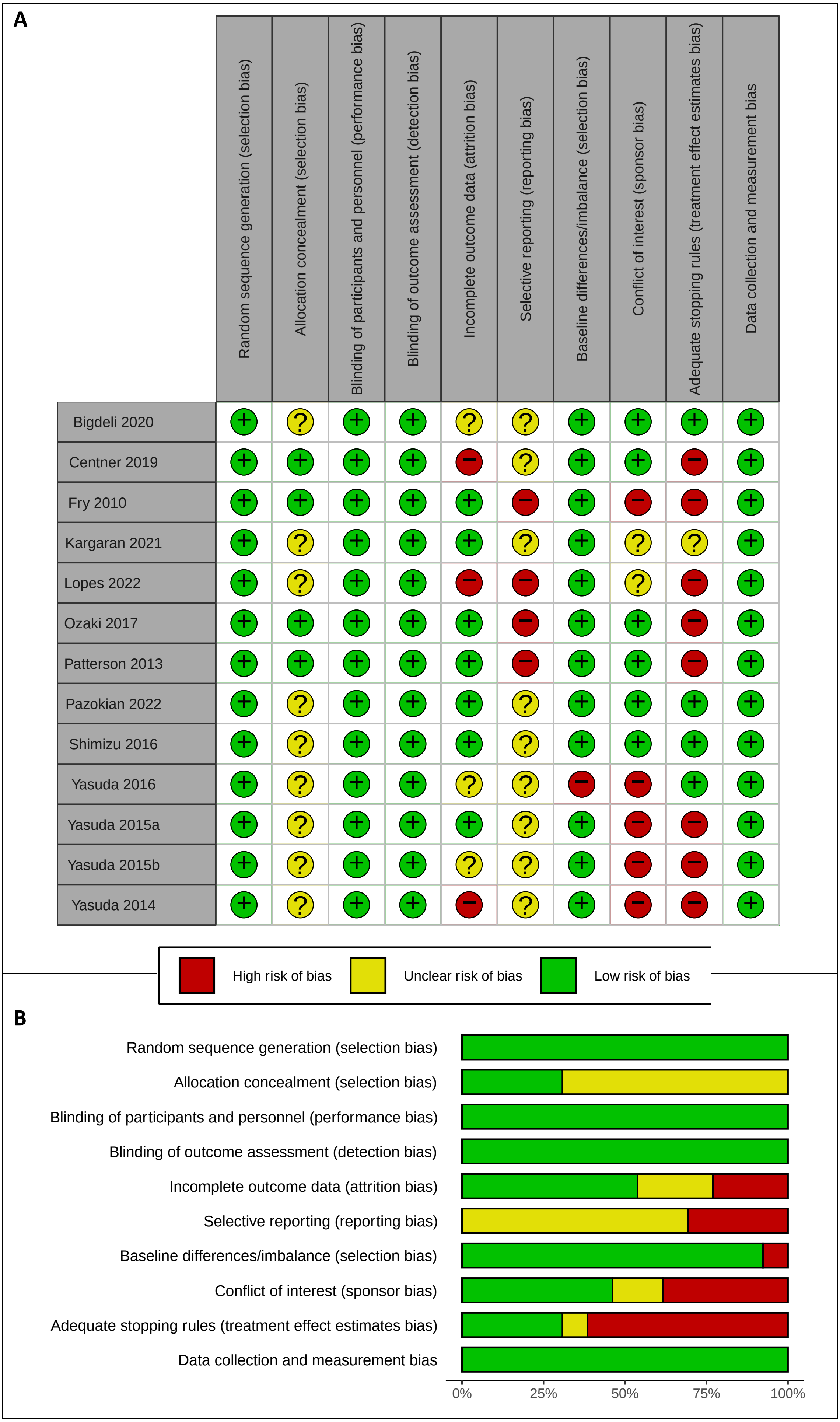

A. Risk of bias summary: review authors' judgements about each Risk of bias item for each included study.

B. Risk of bias graph: review authors' judgements about each Risk of bias item presented as percentages across all included studies. [45–47,49–57]
