# Supplementary material for: Effects of Low-Load Blood Flow Restriction Training on Muscle Anabolism Biomarkers and Thrombotic Biomarkers Compared with Traditional Training in Healthy Adults Older Than 60 Years: Systematic Review and Meta-Analysis
Source: Life (Basel). 2024 Mar 20;14(3):411. doi: 10.3390/life14030411 (PMC10971244; doi:10.3390/life14030411)
Supplement: Supplementary file 1 [file life-14-00411-s001.zip › life-2866246 supplementary/Figure S3. Sensitivity thrombotic risk.pdf]

Figure S3. Sensitivity and publication bias funnel plots for the comparison in thrombotic risk.

LOW-LOAD BFR versus LOW LOAD

A. **Leave-one-out sensitivity analysis**

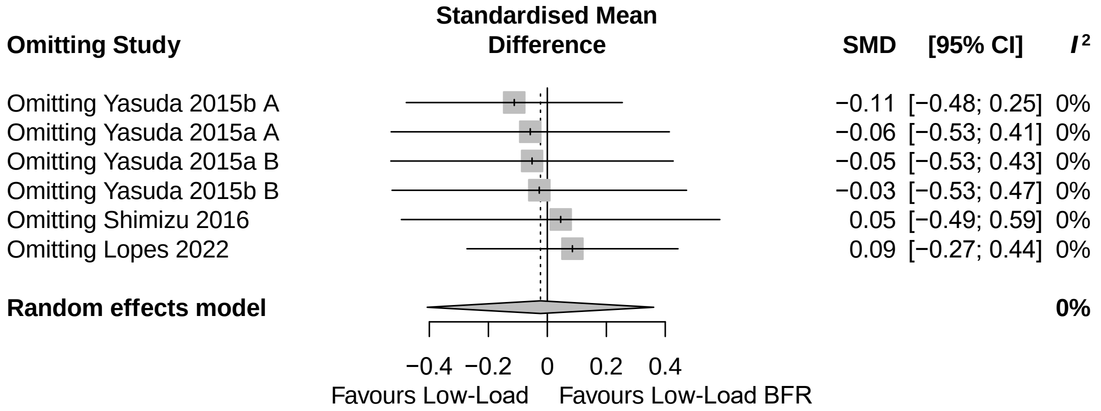

B. **Publication bias**

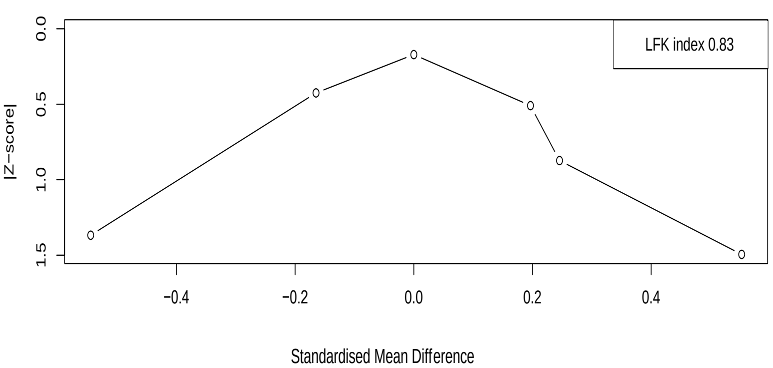

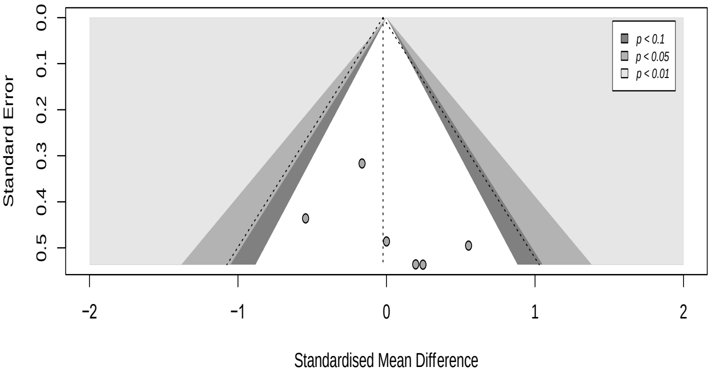

LOW-LOAD BFR versus PASSIVE CONTROL

A. **Leave-one-out sensitivity analysis**

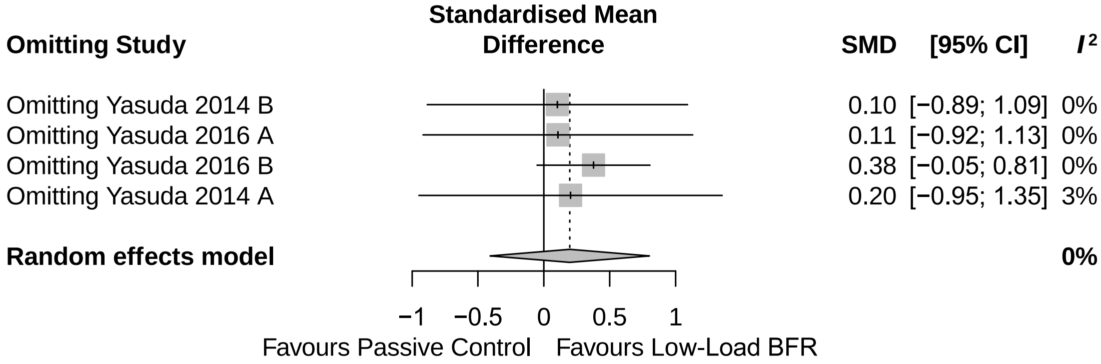

B. **Publication bias**

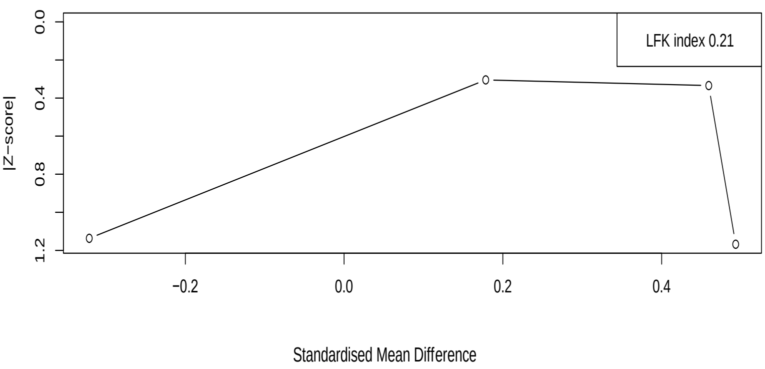

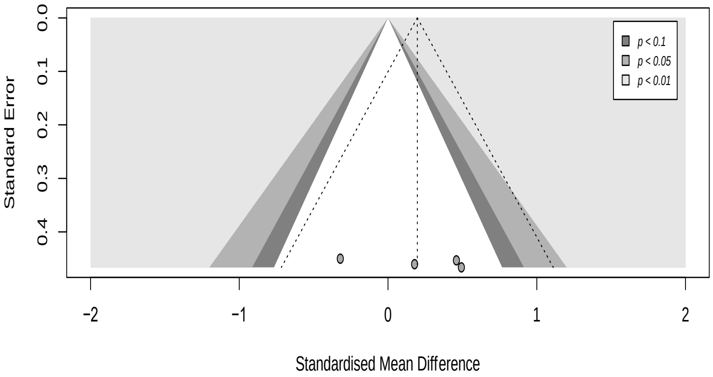

References: [47,48,53,55,56]
