# Supplementary material for: Effects of Low-Load Blood Flow Restriction Training on Muscle Anabolism Biomarkers and Thrombotic Biomarkers Compared with Traditional Training in Healthy Adults Older Than 60 Years: Systematic Review and Meta-Analysis
Source: Life (Basel). 2024 Mar 20;14(3):411. doi: 10.3390/life14030411 (PMC10971244; doi:10.3390/life14030411)
Supplement: Supplementary file 1 [file life-14-00411-s001.zip › life-2866246 supplementary/Scheme S1. Full search strategy ok.pdf]

Scheme S1. Full search strategy

MEDLINE

1. elderly [Title/Abstract] OR "elderly people" [Title/Abstract] OR “Elder”
2. older [Title/Abstract] OR “older adults” [Title/Abstract] OR “old people” [Title/Abstract]
3. aged [Title/Abstract] OR aging [Title/Abstract] OR ageing [Title/Abstract]
4. senior [Title/Abstract] OR geriatric [Title/Abstract]
5. #1 OR #2 OR #3 OR #4
6. "blood flow restriction therapy" [Title/Abstract] OR "blood flow restricted" [Title/Abstract] OR "blood flow-restricted" [Title/Abstract] OR "restricted leg blood flow" [Title/Abstract] OR “restriction training” [Title/Abstract]
7. "vascular occlusion" OR “KAATSU”
8. #6 OR #7
9. ((clinical[Title/Abstract] AND trial[Title/Abstract]) OR clinical trials as topic[MeSH Terms] OR clinical trial[Publication Type] OR random*[Title/Abstract] OR random allocation[MeSH Terms] OR therapeutic use[MeSH Subheading])
10. #5 AND #8 AND #9

WEB OF SCIENCE

1. Topic: elderly OR "elderly people" OR Elder OR older OR "older adults” OR “old people” OR aged OR aging OR ageing OR senior OR geriatric
2. Topic: "blood flow restriction training" OR "blood flow restriction therapy" OR "blood flow restricted" OR "restriction training" OR "occlusion training" OR "vascular occlusion" OR bfrt OR KAATSU

3. Topic: “randomized controlled trial” OR “controlled clinical trial” OR random OR randomly OR trial
4. #1 AND #2 AND #3

Scopus

1. elderly OR "elderly people" OR elder OR older OR "older adults" OR "old people" OR aged OR aging OR ageing OR senior OR geriatric [Article title, abstract, keywords]
2. "blood flow restriction training" OR "blood flow restriction therapy" OR "blood flow restricted" OR "restriction training" OR "occlusion training" OR "vascular occlusion" OR bfrt OR kaatsu [Article title, abstract, keywords]
3. "randomized controlled trial" OR "controlled clinical trial" OR random OR randomly OR trial
4. #1 AND #2 AND #3

Cochrane Controlled Register of Trials (CENTRAL)

1. "blood flow restriction training" OR "blood flow restriction therapy" OR "blood flow restricted" OR "restriction training" OR "occlusion training" OR "vascular occlusion" OR bfrt OR KAATSU [Title, abstract, keyword]
2. elderly OR "elderly people" OR Elder OR older OR "older adults" OR "old people" OR aged OR aging OR ageing OR senior OR geriatric [Title, abstract, keyword]
3. #1 AND #2

PEDro

Abstract & Title: "blood flow restriction" AND "older adults"

Therapy: Strength training

Problem: no selection

Body part: no selection

Subdiscipline: Gerontology

Topic: No selection

Title only: no selection

Method: Clinical Trial

CINAHL

1. elderly or aged or older or elder or geriatric or elderly people or old people or senior
2. "blood flow restriction training" OR "blood flow restriction therapy" OR "blood flow restricted" OR "restriction training" OR "occlusion training" OR "vascular occlusion" OR bfrt OR KAATSU
3. #1 AND #2

Science direct

1. "blood flow restriction training" OR "blood flow restriction therapy" OR "restriction training" OR "occlusion training" OR KAATSU
2. elderly OR aged OR older OR geriatric
3. #1 AND #2
