# Supplementary figures and images for: Effects of Low-Load Blood Flow Restriction Training on Muscle Anabolism Biomarkers and Thrombotic Biomarkers Compared with Traditional Training in Healthy Adults Older Than 60 Years: Systematic Review and Meta-Analysis
Source: Life (Basel). 2024 Mar 20;14(3):411. doi: 10.3390/life14030411 (PMC10971244; doi:10.3390/life14030411)

## LOW-LOAD BFR versus LOW LOAD

## Standardised Mean

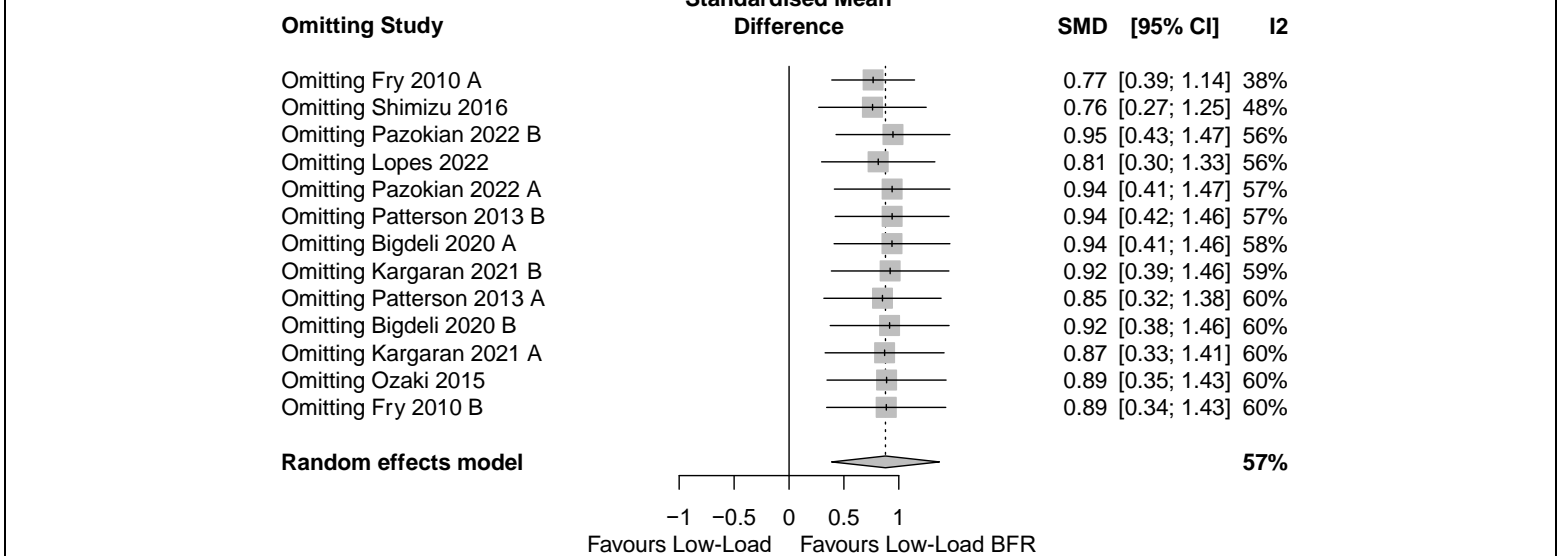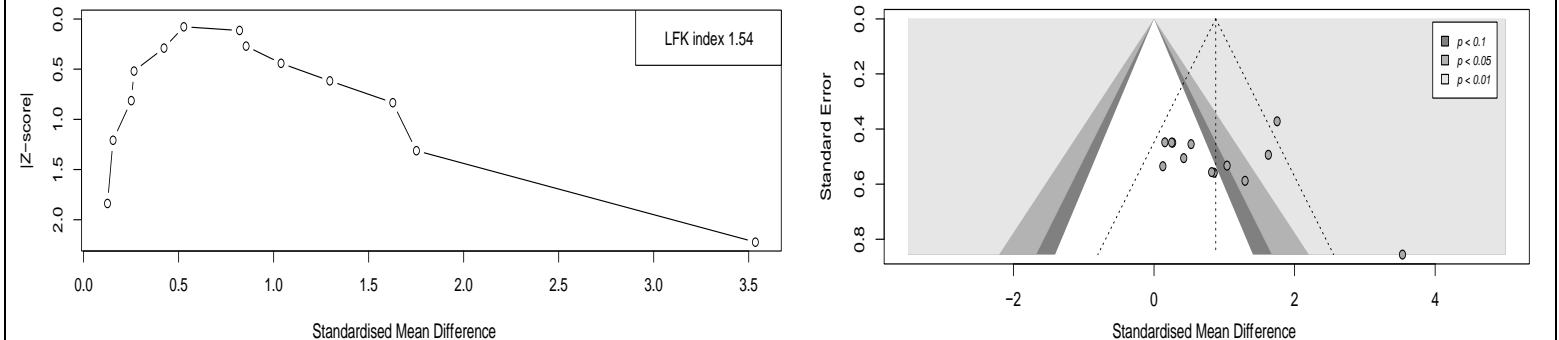

## Standardized Mean

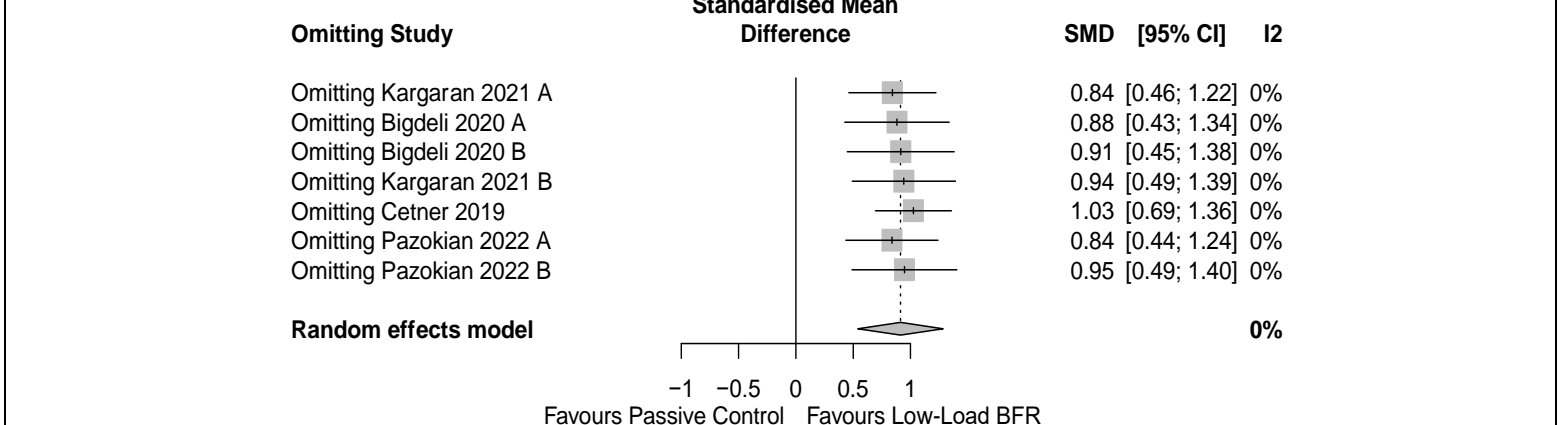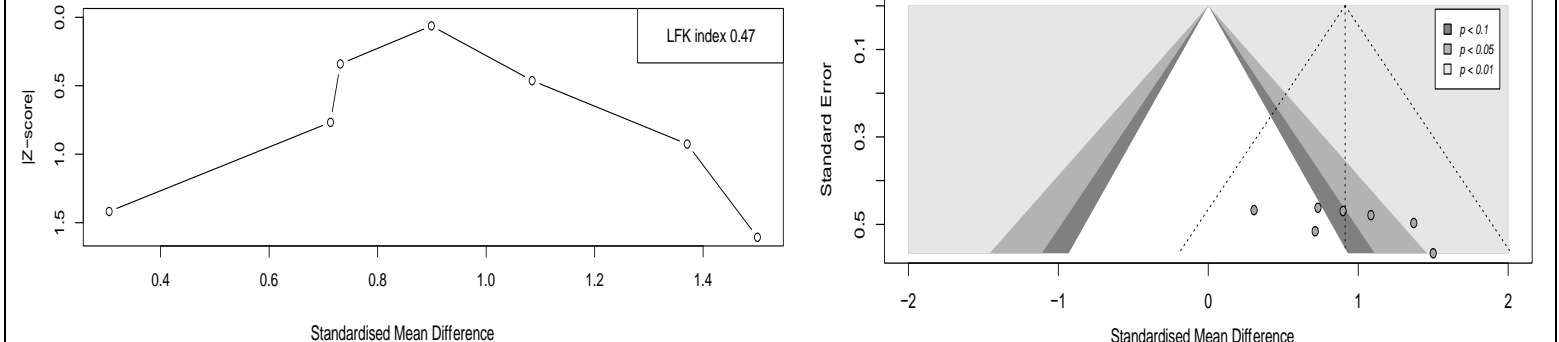

Supplement: Supplementary file 1 [file life-14-00411-s001.zip › life-2866246 supplementary/Figure S2. Sensitivity anabolism.pdf]
